# Implications of sedation during the use of noninvasive ventilation in children with acute respiratory failure (SEDANIV Study)

**DOI:** 10.1186/s13054-024-04976-2

**Published:** 2024-07-11

**Authors:** Lorena Bermúdez-Barrezueta, Juan Mayordomo-Colunga, María Miñambres-Rodríguez, Susana Reyes, Juan Valencia-Ramos, Yolanda Margarita Lopez-Fernandez, Mikel Mendizábal-Diez, Ana Vivanco-Allende, Alba Palacios-Cuesta, Lidia Oviedo-Melgares, José Luis Unzueta-Roch, Jorge López-González, María Teresa Jiménez-Villalta, Maite Cuervas-Mons Tejedor, Lourdes Artacho González, Ainhoa Jiménez Olmos, Martí Pons-Òdena, SEDANIV Study group of the Spanish Society of Pediatric Intensive Care, Marta Brezmes Raposo, María Asunción Pino Vázquez, Ana Vivanco-Allende, Juan Mayordomo-Colunga, María Miñambres-Rodríguez, Susana Beatriz Reyes-Domínguez, Yolanda López Fernández, Zaloa Gorostizaga, María Ángeles García Teresa, María Teresa Rives Ferreiro, Sarah N. Fernández-Lafever, José Manuel González-Gómez, Raúl Montero-Yéboles, Vicente Modesto i Alapont, Antonio Rodríguez-Núñez, Soraya Gutiérrez-Marqués, Aida González-Benavides, Sira Fernández de Miguel, Elcira González-Salas

**Affiliations:** 1 Pediatric and Neonatal Intensive Care, Department of Pediatrics, Hospital Clínico Universitario de Valladolid, Av. Ramón y Cajal, 3, 47003 Valladolid, Spain; 2 Department of Pediatrics, Faculty of Medicine, Valladolid University, Valladolid, Spain; 3 Pediatric Intensive Care Unit, Hospital Universitario Central de Asturias, Oviedo, Spain; 4 Department of Pediatrics, University of Oviedo, Oviedo, Spain; 5 Centro de Investigación Biomédica en Red Enfermedades Respiratorias (CIBERES), Instituto de Salud Carlos III, Madrid, Spain; 6 Primary Care Interventions to Prevent Maternal and Child Chronic Diseases of Perinatal and Developmental Origin (RICORS), Instituto de Salud Carlos III, RD21/0012/0020, Madrid, Spain; 7 Pediatric Intensive Care Unit, Department of Pediatrics, Hospital Universitario Virgen de la Arrixaca, Murcia, Spain; 8 Pediatric Intensive Care Unit, Department of Pediatrics, Complejo Asistencial Universitario de Burgos, Burgos, Spain; 9 Ciencias de la Salud, University of Burgos, Burgos, Spain; 10 Pediatric Intensive Care Unit, Department of Pediatrics, Hospital Universitario de Cruces, BioBizkaia-Bizkaia Health Research Institute, Bizkaia, Spain; 11 Pediatric Intensive Care Unit, Department of Pediatrics, Hospital Universitario de Navarra, Pamplona, Spain; 12 Instituto de Investigación Sanitaria del Principado de Asturias, Oviedo, Spain; 13 Pediatric Intensive Care Unit, Department of Pediatrics, Hospital Universitario, 12 de Octubre, Madrid, Spain; 14 Pediatric Intensive Care Unit, Department of Pediatrics, Hospital Infantil Universitario Niño Jesús, Madrid, Spain; 15 Pediatric Intensive Care Unit Department, Hospital General Universitario Gregorio Marañón, Madrid, Spain; 16 Pediatric Intensive Care Unit, Department of Pediatrics, Hospital Universitario Reina Sofía, Córdoba, Spain; 17 Pediatric Intensive Care Unit, Department of Pediatrics, Hospital Regional Universitario de Málaga, Málaga, Spain; 18 Pediatric Intensive Care Unit, Department of Pediatrics, Hospital Universitario Miguel Servet, Zaragoza, Spain; 19 Inmune and Respiratory Dysfunction Research Group, Institut de Recerca Sant Joan de Déu, Santa Rosa 39-57, 08950 Esplugues de Llobregat, Spain; 20 Pediatric Intensive Care and Intermediate Care Department, Hospital Universitario Sant Joan de Déu, Universitat de Barcelona, Esplugues de Llobregat, Spain

**Keywords:** Sedation, Noninvasive ventilation, Acute respiratory failure, Comfort

## Abstract

**Background:**

The objective of this study was to analyze the effects of sedation administration on clinical parameters, comfort status, intubation requirements, and the pediatric intensive care unit (PICU) length of stay (LOS) in children with acute respiratory failure (ARF) receiving noninvasive ventilation (NIV).

**Methods:**

Thirteen PICUs in Spain participated in a prospective, multicenter, observational trial from January to December 2021. Children with ARF under the age of five who were receiving NIV were included. Clinical information and comfort levels were documented at the time of NIV initiation, as well as at 3, 6, 12, 24, and 48 h. The COMFORT-behavior (COMFORT-B) scale was used to assess the patients’ level of comfort. NIV failure was considered to be a requirement for endotracheal intubation.

**Results:**

A total of 457 patients were included, with a median age of 3.3 months (IQR 1.3–16.1). Two hundred and thirteen children (46.6%) received sedation (sedation group); these patients had a higher heart rate, higher COMFORT-B score, and lower SpO_2_/FiO_2_ ratio than did those who did not receive sedation (non-sedation group). A significantly greater improvement in the COMFORT-B score at 3, 6, 12, and 24 h, heart rate at 6 and 12 h, and SpO_2_/FiO_2_ ratio at 6 h was observed in the sedation group. Overall, the NIV success rate was 95.6%-intubation was required in 6.1% of the sedation group and in 2.9% of the other group (*p* = 0.092). Multivariate analysis revealed that the PRISM III score at NIV initiation (OR 1.408; 95% CI 1.230–1.611) and respiratory rate at 3 h (OR 1.043; 95% CI 1.009–1.079) were found to be independent predictors of NIV failure. The PICU LOS was correlated with weight, PRISM III score, respiratory rate at 12 h, SpO_2_ at 3 h, FiO_2_ at 12 h, NIV failure and NIV duration. Sedation use was not found to be independently related to NIV failure or to the PICU LOS.

**Conclusions:**

Sedation use may be useful in children with ARF treated with NIV, as it seems to improve clinical parameters and comfort status but may not increase the NIV failure rate or PICU LOS, even though sedated children were more severe at technique initiation in the present sample.

**Supplementary Information:**

The online version contains supplementary material available at 10.1186/s13054-024-04976-2.

## Background

Noninvasive ventilation (NIV) is a technique used to support spontaneous breathing. Currently, the best alternative for managing acute respiratory failure (ARF) is invasive mechanical ventilation (IMV), which can cause potential complications. Moreover, its effectiveness in children has been widely demonstrated [1–8]. During the use of NIV, patient adaptation to this kind of respiratory support should be optimal to achieve maximum effectiveness, thus constituting a critical determinant of NIV success [9–11].

The administration of sedative drugs is sometimes used to achieve proper patient adaptation to ventilator and can help reduce anxiety, discomfort and improve tolerance to NIV [10, 12–14]. However, sedatives produce a decreased level of consciousness, the intensity of which depends on the drug, dose used, and individual variability. They may also cause airway obstruction, hypoventilation, apnea, and cardiac depression [15]. Although sedation is currently commonly used during NIV [3, 6], its indications, usefulness, and safety have not been clearly determined, and there are no published studies that analyze the effects of its administration in children. The main objective of this study was to analyze the evolution of clinical parameters and comfort status during the use of NIV depending on whether sedation was administered or not. As a secondary objective, we aimed to determine whether the use of sedatives is correlated with the need for intubation and length of stay (LOS) in the pediatric intensive care unit (PICU) in children with ARF treated with NIV.

## Materials and methods

An observational, prospective, multicenter study was performed, with the initial collaboration of 16 Spanish PICUs. The study period was from January 1st, 2021, to December 31st, 2021. Three PICUs did not complete the protocol or duration of the study, ultimately yielding 13 participating centers (Supplementary material 1).

Children under 5 years of age who were admitted to the PICU, met the clinical criteria for ARF and were treated with NIV for at least 2 h were consecutively included. ARF was defined as the inability of the respiratory system to carry out sufficient gas exchange to meet the metabolic needs of the body, giving rise to ventilation and/or oxygenation disorders [3, 16].

Patients who required intubation within the first 2 h of starting NIV, those on home NIV, postextubation patients, and those who presented any contraindications to starting NIV, such as cardiorespiratory arrest, imminent respiratory exhaustion, hemodynamic instability requiring inotropic support, severe arrhythmias, Glasgow < 9, facial trauma, vocal cord paralysis, undrained pneumothorax, or severe acute respiratory distress syndrome (ARDS) with an SpO_2_/FiO_2_ (S/F) ratio (oxygen saturation [SpO_2_]/fraction of inspired oxygen [FiO_2_]) less than 150, were excluded [4].

### Protocol

NIV was initiated at the discretion of the responsible physician if any of the following conditions were present: ARF without improvement despite medical treatment or another type of respiratory support, progressive dyspnea, hypercapnia with acidosis, or apnea, in the absence of a contraindication for NIV (these were the exclusion criteria).

The choice of NIV interface and modality (continuous positive airway pressure [CPAP] or bilevel positive airway pressure [BLPAP]) was determined by the physician responsible for the patient. Active humidification was used in all the cases. Continuous monitoring was performed via electrocardiography, pulse oximetry, and respiratory rate. Clinical monitoring was also carried out with the Modified Wood Clinical Asthma Scale (mWCAS) [17]. Additionally, blood gas analyses were performed independently of the study.

The patients’ well-being was determined with the COMFORT behavior (COMFORT-B) scale, which includes the “crying” category [18]. The score on this scale ranges from 6 to 30 points (6–10, very comfortable; 11–22, comfortable; and 23–30, not at all comfortable) [19]. Sedation was administered at the discretion of the responsible physician or according to the protocol of each PICU.

### Data collection

Physiological data, clinical data, and ventilator parameters were recorded at the time of NIV initiation and again at 3, 6, 12, 24, and 48 h after NIV commenced. The data recorded included heart rate, respiratory rate, SpO_2_, FiO_2_, S/F ratio, mWCAS and COMFORT-B score, NIV modality (CPAP or BLPAP), interface, inspiratory positive airway pressure (IPAP), expiratory positive airway pressure (EPAP), pH and pCO_2_ if arterial, capillary or venous blood gas measurements were available, enteral nutrition and nonpharmacological comfort measures. When calculating the S/F ratio, patients with cyanotic congenital heart disease and SpO_2_ values > 97% were excluded since the SpO_2_–PaO_2_ correlation is lost above this value [20, 21].

If sedation was used, the reason, drug, dose, timing, route and method of administration were recorded, as were any adverse events. Adverse events were considered to be those apparently related to sedation requiring some intervention such as interrupting or decreasing sedation, increasing respiratory support, fluid or vasopressor administration. Potential adverse events included: bradycardia, defined as a heart rate at the lower limit of normal (2nd percentile) for age [22]; hypotension, defined as a systolic blood pressure of less than the 5th percentile derived from normative data for age, sex, and height [23, 24]; respiratory depression or apnea (ineffective respiratory effort, oxygen desaturation).

Data on weight, age, sex, comorbidities, PRISM (Pediatric Risk of Mortality) III score at the start of NIV and at 24 h, etiological diagnosis of ARF, need for intubation, days of stay in the PICU, and mortality were also recorded.

### Clinical outcomes

The effect of the administration of sedation on the following outcomes was assessed:

1. Changes in physiological parameters (heart rate, respiratory rate), COMFORT-B score, mWCAS, and the SpO_2_/ FiO_2_ ratio at 3, 6, 12, 24, and 48 h. The numerical difference between the values recorded at the specified time points and the initial values was analyzed.
2. NIV failure, which was defined as the need for intubation during the use of the technique according to the physician in charge decision. The suggested failure criteria and possible reason for intubation were: clinical symptoms of severe respiratory distress with signs of imminent respiratory exhaustion, persistent apneas, altered state of consciousness, need for a FiO_2_ above 0.6 to keep the SpO_2_ above 90% despite NIV optimization, and hypercapnia with a pH < 7.20.
3. Days of stay in the PICU.

### Statistical analyses

Categorical data are expressed as absolute values and percentages. For quantitative data, the mean and standard deviation (SD) were used; otherwise, the median and interquartile range (IQR) were used if the data were not normally distributed. For the analysis of continuous variables, the Mann‒Whitney U test or Student’s t test was used; for categorical variables, Fisher’s exact test or the chi-square test was used, depending on the normality of the distribution of the sample.

To evaluate the effect of sedation on NIV failure, univariate and multivariate logistic regression analyses were performed using the backward stepwise method based on the likelihood ratio (LR). The results are presented as odds ratios (ORs) and 95% confidence intervals (CIs). Receiver operating characteristic (ROC) curves were used to determine cutoff values for the predictive models obtained. The area under the ROC curve (AUC) and the log likelihood (-2LL) were used as measures of predictive ability.

The effect of sedation on the LOS in the PICU was evaluated with univariate Cox proportional hazards regression models and multivariate regression models using the backward stepwise method based on the LR. An event was defined as “PICU discharge”, assessed by the speed at which the event occurred. The results are expressed as hazard ratios (HRs) and corresponding 95% CIs, with an HR < 1 indicating a slower speed and thus a longer stay in the PICU.

To construct the multivariate models, variables with a *p* value < 0.1 in the univariate analysis and those with clinical relevance described in previous studies as predictors of NIV failure or longer PICU LOS were included [3–5, 25]. If there was a risk of collinearity, the earliest variable was selected. A value of *p* < 0.05 was considered to indicate statistical significance.

### Ethical considerations

This study was authorized by the Spanish Agency of Medicines and Medical Devices as an observational postauthorization study (code LBB-MOR-2020-01) and was approved by the Drug Research Ethics Committee of the East Valladolid Health Department (internal code 20-1954) in accordance with the regulations of the Declaration of Helsinki. The Ethics Committee of each participating institution approved the protocol, and the need for informed consent was in line with local regulations.

## Results

**Fig. 1:**
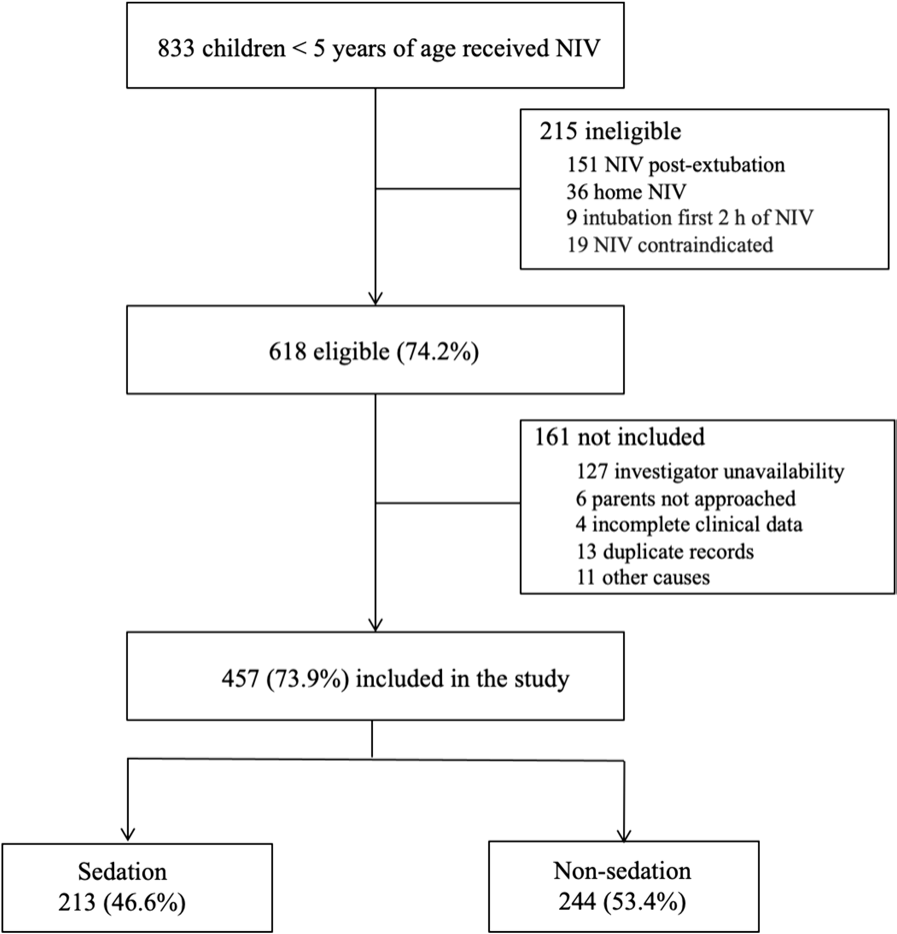
Flowchart of the study population. *NIV,* noninvasive ventilation

A total of 457 patients were included during the study period (Fig. 1). The median age was 3.3 months (IQR 1.3–16.1), and the median weight was 5.8 kg (IQR 4–10). The main cause of ARF was acute bronchiolitis (60.8%). The BLPAP modality was used as the first line of treatment in 79% of patients, and the total face mask was the most commonly used interface (82.9%). Table 1 describes the baseline characteristics of the patients according to sedation status.

**Table 1:** Demographic and clinical characteristics of the patients at baseline according to sedation status

	Total n = 457 (%)	Non-Sedation N = 244 (%)	Sedation 213 (%)	*p-*value
Age (months); median [IQR]	3.3 [1.3–16.1]	3 [1.3–19.8]	3.4 [1.3–12.5]	0.375
Age group (months)				0.016
1–3	223 (48.8)	122 (50)	101 (47.4)	
3–12	97 (21.2)	40 (16.4)	57 (26.8)	
> 12	137 (30)	82 (33.6)	55 (25.8)	
Weight (kg); median [IQR]	5.8 [4–10]	5.5 [4–10.7]	6 [4–9.3]	0.552
Males; n (%)	250 (54.7)	129 (52.9)	121 (56.8)	0.339
Prematurity; n (%)	82 (17.9)	41 (16.8)	41(19.2)	0.510
Patients with at least 1 comorbidity; n (%)	80 (17.5)	41 (18.8)	39 (18.3)	0.663
^a^Underlying disease; n (%)				
Non-cyanotic cardiopathy	25 (5.5)	13 (5.3)	17 (8)	0.253
Cyanotic cardiopathy	5 (1.1)	2 (0.8)	3 (1.4)	0.668
Bronchopulmonary dysplasia	19 (4.2)	11 (4.5)	8 (3.8)	0.688
Neuromuscular disease	16 (3.5)	11 (4.5)	5 (2.3)	0.210
Congenital malformation syndromes	13 (2.8)	7 (2.9)	6 (2.8)	1
Down's syndrome	6 (1.3)	3 (1.2)	3 (1.4)	1
Immunodeficiency	4 (0.9)	3 (1.2)	1 (0.5)	0.627
Other	16 (3.5)	10 (4.1)	6 (2.8)	0.457
Diagnosis; n (%)				0.491
Bronchiolitis	278 (60.8)	148 (60.7)	130 (61)	
Bronchospasm	93 (20.4)	46 (18.9)	47 (22.1)	
Pneumonia	44 (9.6)	27 (11.1)	17 (8)	
Cardiogenic pulmonary edema	11 (2.4)	4 (1.6)	7 (3.3)	
Sepsis	15 (3.3)	8 (3.3)	7 (3.3)	
Other	16 (3.5)	11 (4.5)	5 (2.3)	
ARDS; n (%)	6 (1.3)	1 (0.4)	5 (2.3)	0.102
PRISM III score at NIV initiation; mean (SD)	1.75 (2.5)	1.40 (2.3)	2.16 (2.8)	0.001
PRISM III score at 24 h; mean (SD)	0.91 (2.1)	0.62 (1.8)	1.25 (2.3)	< 0.001
HFNC prior to NIV; n (%)	203 (44.4)	89 (36.5)	114 (53.3)	< 0.001
Heart rate (beats/min); mean (SD)	166.4 (25.1)	162.5 (25.2)	170.8 (24.2)	< 0.001
Heart rate by age group; mean (SD)				
0–3 mo	171.4 (21.9)	170.9 (21.9)	172.1 (25.6)	0.671
3–12 mo	171.2 (27.5)	163.8 (28.7)	176.3 (25.6)	0.031
> 12 mo	155 (24.4)	149.6 (22.9)	162.6 (24.7)	0.002
Respiratory rate (breaths/min); median [IQR]	51 [43–64]	50 [40–60]	55 [46–65]	< 0.001
Respiratory rate by age group; median [IQR]				
0–3 mo	54 [45–65]	50 [43–62]	55 [46–66]	0.089
3–12 mo	54 [45–65]	53 [45–66]	58 [45–65]	0.696
> 12 mo	48 [40–60]	45 [37–55]	51 [45–62]	0.002
FiO_2_ (%); median [IQR]	40 [30–50]	35 [30–45]	40 [30–50]	0.233
Oxygen saturation (SpO_2_) %; median [IQR]	97 [95–99]	97 [95–99]	97 [95–99]	0.685
^b^S/F ratio; median [IQR]; n = 244	243 [192–323]	254 [216–323]	243 [180–323]	0.273
mWCAS; median [IQR]; n = 388	6.1 (2)	5.7 (2)	6.5 (2.1)	0.001
COMFORT-B scale; median [IQR]; n = 374	22 [18–24]	20 [16–23]	23 [20–25]	< 0.001
pH; median [IQR]; n = 285	7.33 [7.27–7.38]	7.34 [7.27–7.39]	7.32 [7.27–7.37]	0.028
pCO_2_ (mmHg); median [IQR]; n = 285	47.3 [39–61]	46.9 [38.8–59.7]	50 [42–63]	0.077
Mode of ventilation at NIV initiation; n (%)				0.772
CPAP	96 (21)	50 (20.5)	46 (21.6)	
Bi-level pressure (BLPAP)	361 (79)	194 (79.5)	167 (78.4)	
Interface; n (%)				0.592
Total face mask	379 (82.9)	206 (84.4)	173 (81.2)	
Nasal mask	45 (9.9)	20 (8.2)	25 (11.7)	
Nasal cannula	30 (6.6)	17 (7)	13 (6.1)	
Oronasal mask	2 (0.4)	1 (0.4)	1 (0.5)	
Helmet	1 (0.2)	0	1 (0.5)	

^a^Some patients present with more than one condition

^b^Patients with SpO_2_ > 97% and those with a cyanotic cardiopathy were excluded for the calculation of the S/F ratio

Categorical variables are expressed as absolute value and percentage (%). Quantitative variables are expressed as mean and standard deviation (SD) or median and interquartile range [IQR] if they were not normally distributed

*ARDS:* acute respiratory distress syndrome; *HFNC:* high-flow nasal cannula oxygen therapy; *PRISM III score:* Pediatric Risk of Mortality Score III; *mWCAS:* modified Wood’s Clinical Asthma Score; *S/F ratio:* SpO_2_/FiO_2_ ratio

### Sedative agents

Sedation was used in 213 children (46.6%; 95% CI 41.9–51.3) (sedation group); in 79 (37%) children, it was administered at the beginning of NIV, and 122 of the patients (57.3%) had received some form of sedation 3 h after the start of NIV. Benzodiazepines were the most commonly used drugs (47.9%), followed by alpha-2 agonists (35.7%), as shown in Table 2. Midazolam was the preferred benzodiazepine and was administered intermittently in 15.5% of the children, with a median of 2 intravenous (IV) boluses (IQR 1–3), a median initial dose of 0.1 mg/kg (IQR 0.07–0.1), and a median cumulative dose of 0.16 mg/kg (IQR 0.1–0.3). In 8.9% of the children, it was provided as a continuous IV infusion with a median initial dose of 0.08 mg/kg/h (IQR 0.05–0.1) and a median cumulative dose of 1.73 mg/kg (IQR 1.2–3.3). Dexmedetomidine as a continuous IV infusion was the most commonly used alpha-2 agonist sedative (23.5%), with a median initial dose of 0.5 µg/kg/h (IQR 0.4–0.5) and a median cumulative dose of 16.5 µg/kg (9.2–33.1). Differences per participating center in rate of sedation use, firs-line agent, and route and method of administration are shown in Supplementary material 2.

**Table 2:** Information on sedation administration in the study population

	n = 213 (%)
Timing of sedative administration	
At NIV initiation	79 (37)
First 3 h of NIV	122 (57.3)
First 6 h of NIV	137 (64.3)
First 12 h of NIV	172 (80.8)
First 24 h of NIV	194 (91.1)
Reason for sedation	
Agitation	103 (48.4)
Patient-ventilator asynchrony	38 (17.8)
At the start of NIV to improve adaptability	24 (11.3)
Work of breathing	5 (2.3)
ªOther reasons	9 (4.2)
Unknown	34 (16)
Route of administration	
Intravenous (IV)	122 (57.3)
Oral route (OR)	74 (34.7)
Intravenous and oral (IV and OR)	17 (8)
Intranasal	1 (0.5)
Methods of sedation	
Intermittent only	128 (60.1)
Continuous IV infusion only	41 (19.2)
Intermittent and continuous IV infusion	44 (20.7)
Hours of continuous IV infusion; *median [IQR]*	33 [21 – 60]
Number of sedatives used per patient	
Only one sedative	152 (71.4)
Two sedatives	48 (22.5)
Three sedatives	13 (6.1)
Sedatives used	
Midazolam	52 (24.4)
Dexmedetomidine	50 (23.5)
Levomepromazine	34 (16)
Clonidine	26 (12.2)
Ketamine	26 (12.2)
Diazepam	23 (10.8)
Lorazepam	19 (8.9)
Propofol	17 (8)
Dipotassium clorazepate	15 (7)
Morphine	14 (6.6)
Chloral hydrate	7 (3.3)
Fentanyl	2 (0.9)
Chlorpromazine	2 (0.9)
First line agent	
Midazolam	44 (20.7)
Dexmedetomidine	33 (15.5)
Clonidine	24 (11.3)
Levomepromazine	20 (8.9)
Ketamine	19 (8.9)
Lorazepam	18 (8.5)
Dipotassium clorazepate	14 (6.6)
Morphine	14 (6.6)
Propofol	13 (6.1)
Diazepam	10 (4.7)
Chloral hydrate	3 (1.4)
Fentanyl	1 (0.5)
Chlorpromazine	1 (0.5)

ªNasogastric tube placement, cannulation of peripheral or central veins, performance of lung ultrasound

*NIV* non-invasive ventilation, *IV* intravenous, *OR* oral route

### Nonpharmacological measures

Nonpharmacological comfort measures were utilized in 309 children (67.6%). These included pacifiers, sucrose, music therapy, holding the patients in caretakers’ arms, or other distraction strategies (such as games or playing on tablets). These measures were used less frequently in the sedation group, albeit with no significant differences in comparison with children who did not receive sedatives (65.7% vs. 70.7%; *p* = 0.255).

### Enteral nutrition

At 12 h, 56.3% of patients had started enteral feeding. Seventy children (15.3%) remained in a fasting state during the first 48 h, 166 (36.3%) tolerated partial enteral nutrition, and 214 (46.8%) achieved full enteral nutrition. At 48 h, patients who were partially or fully fed had better COMFORT-B score [mean 15.1 (SD 3.7) vs. 17.2 (3.5); *p* = 0.028]. No differences were found in feeding patterns with or without the use of sedation.

### Adverse effects

Adverse effects associated with sedation were recorded in 8% of patients: 13 presented with bradycardia without hemodynamic repercussion (11 with dexmedetomidine, 2 with midazolam), and 4 presented with respiratory depression (3 with benzodiazepines and 1 with propofol), requiring a temporary increase in noninvasive respiratory support and interruption or reduction of continuous IV infusion. No intubation seemed to be related to the use of sedation according to the local investigators.

### Clinical outcomes

#### Sedation use and physiological parameters

**Fig. 2:**
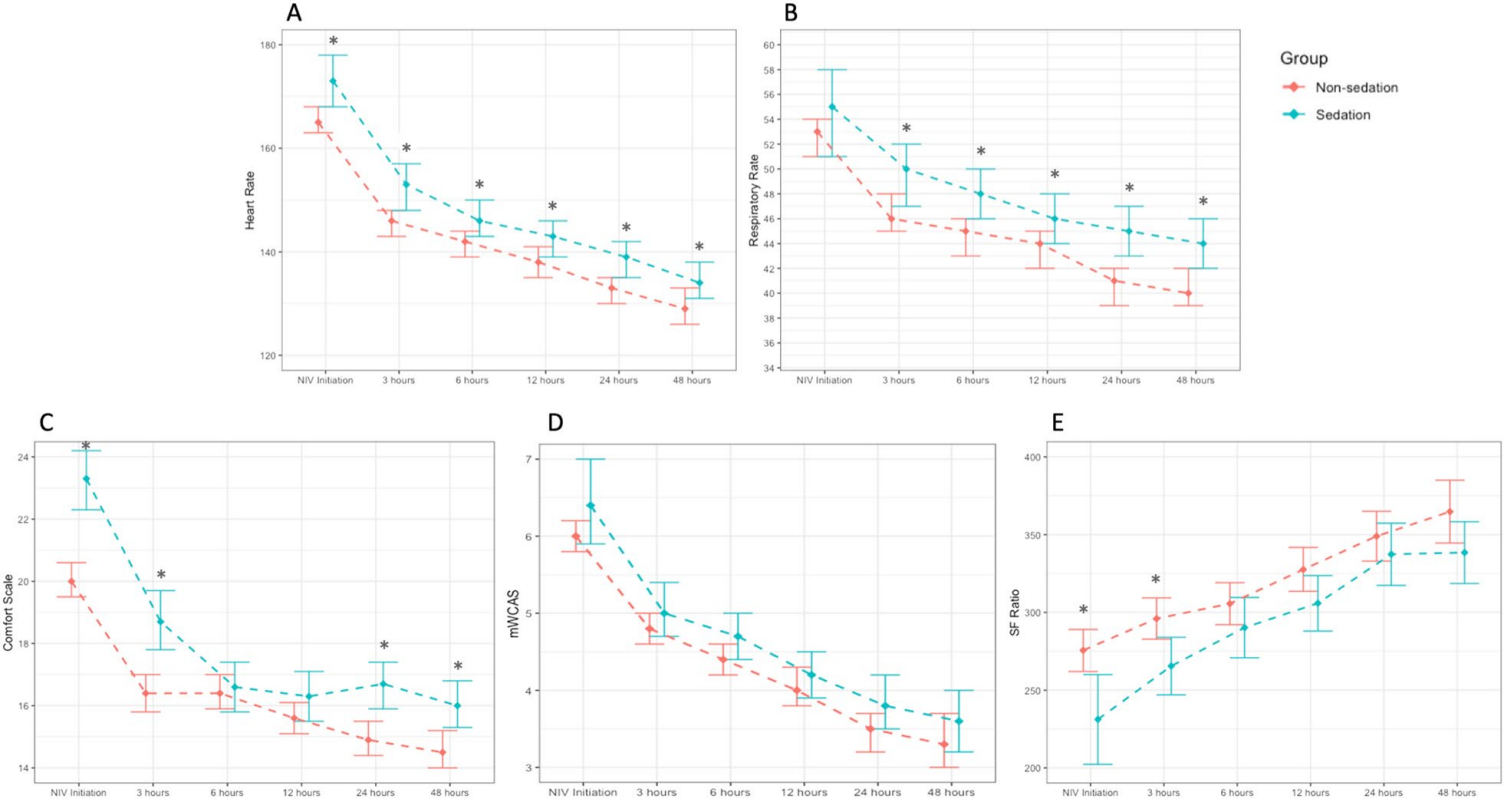
The data were collected at 0, 3, 6, 12, 24 and 48 h after NIV initiation according to sedation status. The mean and 95% confidence intervals are shown. **A** Heart rate. **B** Respiratory rate. **C** Comfort-B scale. (D) SpO_2_/FiO_2_ (S/F) ratio. (E) Modified Wood’s clinical asthma score. ªSpO_2_ over 97% was excluded from the calculation of the S/F ratio. *p < 0.05 for between-group comparisons

At NIV initiation, sedation group children had higher PRISM scores (2.2 ± 2.8 vs 1.40 ± 2.3; *p* = 0.001), more tachycardia (173 ± 24 vs. 165 ± 25; *p* = 0.014), more distress [COMFORT-B score (23.3 ± 3.8 vs. 20 ± 4.7; *p* < 0.001)], and more hypoxemia [S/F ratio (231 ± 100.3 vs. 276 ± 95.6; *p* = 0.002)], showing no statistically significant differences in respiratory rate (55 ± 14.8 vs. 53 ± 14; *p* = 0.403) and mWCAS (6.4 ± 2.2 vs. 6 ± 2; *p* = 0.321) (Fig. 2). The five parameters (heart rate, COMFORT-B score, S/F ratio, respiratory rate and mWCAS) showed progressive improvement during NIV treatment in both the sedation and non-sedation groups, although a significantly greater change was observed in the COMFORT-B scale at 3, 6, 12, and 24 h; in the heart rate at 6 and 12 h; in the mWCAS at 3 and 12 h; and in the S/F ratio at 6 h in the group that received sedation (Table 3).

**Table 3:** Changes of heart rate, respiratory rate, COMFORT-B scale, mWCAS and S/F ratio assessed at different moments during NIV comparing children who received sedatives VS. those who did not. Note that the sample size of non-sedation and sedation groups varies according to the time studied

	Non-Sedation	Sedation	*p-*value
At 3 h; n = 457	n = 335	n = 122	
Heart rate decrease; mean (SD)	− 18.3 (22.4)	− 21.4 (22.9)	0.160
Respiratory rate decrease; mean (SD)	− 6.3 (12.4)	− 5.8 (13.3)	0.595
COMFORT-B scale decrease; mean (SD); n = 355	− 3.3 (4.4)	− 4.6 (5.1)	0.030
mWCAS; mean (SD)	− 1.1 (1.2)	− 1.5 (1.5)	0.036
S/F ratio increase; mean (SD); n = 179	13.2 (50.3)	23.4 (53.2)	0.177
At 6 h; n = 451	n = 313	n = 138	
Heart rate decrease; mean (SD)	− 21.5 (24.3)	− 27 (24.6)	0.033
Respiratory rate decrease; mean (SD)	− 6.8 (14.2)	− 8.8 (13.7)	0.106
COMFORT-B scale decrease; mean (SD); n = 348	− 3.2 (4.6)	− 6.5 (4.9)	< 0.001
mWCAS; mean (SD)	− 1.5 (1.4)	− 1.8 (1.6)	0.112
S/F ratio increase; mean (SD); n = 166	23.1 (71.1)	40.5 (55.4)	0.069
At 12 h; n = 435	n = 268	n = 167	
Heart rate decrease; mean (SD)	− 24.6 (25.8)	− 30.4 (25.3)	0.023
Respiratory rate decrease; mean (SD)	− 7.8 (13.7)	− 9.8 (13.9)	0.160
COMFORT-B scale decrease; mean (SD); n = 328	− 4.1 (4.8)	− 6.3 (5.4)	< 0.001
mWCAS; mean (SD)	− 1.8 (1.7)	− 2.2 (1.8)	0.050
S/F ratio increase; mean (SD); n = 158	50.6 (70.5)	48.6 (74.9)	0.791
At 24 h; n = 385	n = 220	n = 165	
Heart rate decrease; mean (SD)	− 31.4 (24.7)	− 34 (28)	0.356
Respiratory rate decrease; mean (SD)	− 10.9 (13.6)	− 10.8 (14.3)	0.952
COMFORT-B scale decrease; mean (SD); n = 283	− 4.8 (5.2)	− 6.1 (5.4)	0.042
mWCAS; mean (SD)	− 2.3 (1.8)	− 2.6 (2.1)	0.132
S/F ratio increase; mean (SD); n = 134	75.1 (92.9)	80.4 (91.4)	0.851
At 48 h; n = 267	n = 145	n = 122	
Heart rate decrease; mean (SD)	− 37 (23.2)	− 35.4 (29)	0.625
Respiratory rate decrease; mean (SD)	− 12.8 (12.9)	− 12.4 (16.9)	0.829
COMFORT-B scale decrease; mean (SD); n = 208	− 5 (5.3)	− 6.2 (4.9)	0.098
mWCAS; mean (SD)	− 2.5 (1.9)	− 2.9 (2)	0.124
S/F ratio increase; mean (SD); n = 167	84.7 (92.8)	83.2 (93.8)	0.938
Whole sample	n = 244	n = 213	
NIV failure	7/244 (2.9)	13/212 (6.1)	0.092
Duration of NIV (hours); median [IQR]	56.5 [30.2–92.8]	59 [33.2–99]	0.213
PICU LOS (days); median [IQR]	4 [3–6]	5 [3–8]	0.019

*mWCAS* modified Wood’s Clinical Asthma Score, *S/F ratio* SpO_2_/FiO_2_ ratio*, PICU LOS* length of stay in the Pediatric Intensive Care Unit

Categorical variables are expressed as absolute value and percentage (%). Quantitative variables are expressed as mean and standard deviation (SD), or median and interquartile range [IQR] if they were not normally distributed. A *p*-value < 0.05 is considered statistically significant

SpO_2_ over 97% were excluded to calculate the S/F ratio

#### Sedation and NIV failure

Twenty-two patients were intubated. In 20 children, NIV failed, and they required intubation during the first 72 h (4.4%, 95% CI 2.4–6.4). The median time to intubation was 23 h (IQR 7.4–46.9). The remaining two children were intubated at 7 and 8 days, respectively, due to a new condition other than the cause of ARF that led to NIV being started; therefore, these cases were not attributed to treatment failure. The reasons for failure were hypoxemia (10), hypercapnia (4), fatigue (3), hemodynamic instability (2), and apnea (1).

Sedative use was more frequent in patients who failed the NIV trial (65% vs. 45.8%; *p* = 0.092). Univariate analysis of the data revealed that among children who required intubation, those with underlying disease, prematurity and a PRISM III score at NIV initiation were significantly more frequent, while the baseline respiratory rate was greater and the SpO_2_ was lower (Table 4). Supplementary material 3 offers information on physiological and ventilation parameters, comparing the success and failure groups.

**Table 4:** Demographic, baseline parameters and sedation status according to the success or failure of NIV. Univariate analyses

	Success group n = 437 (%)	Failure group n = 20 (%)	*p*-value
Patients’ characteristics			
Age (months); median [IQR]	3.3 [1.3–16.2]	3.7 [1–12.3]	0.824
Weight (kg); median [IQR]	6 [4–10]	4.7 [3.4–7.8]	0.091
Males; n (%)	238 (54.5)	12 (60)	0.627
Underlying disease; n (%)	72 (16.5)	8 (40)	0.008
Prematurity; n (%)	75 (17.2)	7 (35)	0.043
PRISM III score at NIV initiation; mean (SD)	1.6 (2.3)	5.5 (4.6)	< 0.001
PRISM III score at 24 h; mean (SD)	0.7 (1.6)	5.4 (4.4)	< 0.001
HFNC prior to NIV; n (%)	198 (45.3)	5 (25)	0.063
Baseline physiological and clinical parameters			
Heart rate (beats/min); mean (SD)	166.2 (24.7)	170.2 (32.3)	0.447
Respiratory rate (breaths/min); mean (SD)	52.7 (13.9)	62.6 (17)	0.019
FiO_2_ (%); median [IQR]	40 [30–50]	43 [30–59]	0.105
SpO_2_ (%); median [IQR]	97 [95–99]	95.5 [90.3–97]	0.006
ªS/F ratio; median [IQR]; n = 244	254.1 [194–323.3]	211 [151.7–310]	0.060
mWCAS; median [IQR]; n = 388	6 [5–7]	7 [6–9]	0.064
COMFORT-B scale; median [IQR]; n = 374	22 [18–24]	23 [17–25]	0.577
Blood gases at NIV initiation; n = 285			
pH	7.33 [7.27–7.38]	7.30 [7.17–7.36]	0.199
pCO_2_ (mmHg)	47 [39–61]	54 [43–65.8]	0.233
Ventilator settings at NIV initiation			
Mode of ventilation; n (%)			
CPAP	94 (21.5)	2 (10)	0.217
Bi-level pressure (BLPAP)	343 (78.5)	18 (90)	
Bi-level pressure (BLPAP); n = 337			
IPAP (cmH_2_O)	10 [9–12]	11 [8–15]	0.239
EPAP (cmH_2_O)	6 [5, 6]	6 [5–8.3]	0.092
CPAP (cmH_2_O); n = 96	5 [5, 6]	6 [6–6]	0.415
Tidal volume per kg of weight (mL); n = 304	8.7 [7–10]	8 [6–10]	0.519
Sedation	200 (45.8)	13 (65)	0.092
Facial mask interface; n (%)	362 (82.8)	17 (85)	0.852
Non-pharmacological measures; n = 452 (%)	294 (68.1)	15 (75)	0.514

*mWCAS* modified Wood’s Clinical Asthma Score, *S/F ratio* SpO_2_/FiO_2_ ratio, *PRISM III score* Pediatric Risk of Mortality Score III, *IQR* interquartile range, *SD* standard deviation, *NIV* noninvasive ventilation, *CPAP* continuous positive airway pressure, *EPAP* expiratory positive airway pressure, *IPAP* inspiratory positive airway pressure

ªSpO_2_ over 97% were excluded to calculate the S/F ratio

Categorical variables are expressed as absolute value and percentage (%). Quantitative variables are expressed as mean and standard deviation (SD), or median and interquartile range [IQR] if they were not normally distributed

A multivariate analysis (Supplementary material 4) was performed on the general sample, and the best predictive model for NIV failure was chosen; this model included the PRISM III score at NIV initiation (OR 1.408; 95% CI 1.230–1.611) and the respiratory rate at 3 h (OR 1.043; 95% CI 1.009–1.079), with a predictive capacity of − 2LL = 129.57 and an AUC of 0.807 (95% CI 0.687–0.928, *p* < 0.001). The optimal cutoff points suggested as predictors of failure were a PRISM III score of 4.2 and a respiratory rate at 3 h of 79 bpm (sensitivity 80% and specificity 81.6%). The use of sedation was not shown to be an independent predictor of NIV failure.

Furthermore, given the known relevance of the S/F ratio as a predictor of NIV failure [4, 21], a second multivariate analysis was performed with a reduced sample of patients (n = 262) to evaluate the effect of sedation adjusted for the S/F ratio. This analysis identified the S/F ratio at 3 h (OR = 0.992; 95% CI = 0.984–0.999) and the PRISM III score at NIV initiation (OR = 1.445; 95% CI = 1.215–1.719) as independent predictors of failure, while sedation was not associated with treatment failure (predictive capacity-2LL = 86.19 and AUC = 0.815; 95% CI = 0.691–0.939, *p* < 0.001). The suggested optimal S/F ratio cutoff point was 180.5 (sensitivity 73.3% and specificity 72%) (Supplementary material 3).

#### Sedation and length of PICU stay

The PICU LOS was significantly greater in patients who received sedation (5 days, IQR 3–8 vs. 4 days, IQR 3–6; *p* = 0.019). Cox regression analysis was used to determine the factors associated with a longer PICU stay (hazard ratio [HR] < 1): weight (HR 1.072, 95% CI 1.041–1.103), PRISM III score at 24 h (HR 0.859, 95% CI 0.803–0.920), respiratory rate at 12 h (HR 1.017, 95% CI 1.006–1.027), SpO_2_ at 3 h (HR 1.069, 95% CI 1.023–1.117), FiO_2_ at 12 h (HR 0.031, 95% CI 0.004–0.219), NIV failure (HR 0.275, 95% CI 0.130–0.580), and hours of NIV (HR 0.995, 95% CI 0.993–0.997). According to the adjusted model, sedation was not related to a longer PICU stay (Supplementary material 4).

Five patients died (1.1%), but none of these deaths were attributable to the use of NIV or sedatives.

## Discussion

The present study suggests that sedation can contribute to improving the physiological parameters and comfort status of children younger than 5 years with ARF during the use of NIV without promoting NIV failure or prolonging their PICU stay. To our knowledge, this is the first study focused on assessing the effects of sedation in children with ARF during NIV.

The prevalence of sedation practices during the use of NIV is highly variable. Pediatric studies that have recorded this information are limited and include a wide range of sedation use, ranging from 12 to 78% [2, 4, 5, 21]. In our cohort, almost half of the patients received sedatives, preferably intermittently, during the first hours of NIV, the two most common of which were midazolam and dexmedetomidine. The findings of the only international survey on sedation practices during the use of NIV, directed at adult patients, showed that benzodiazepines were the most commonly used drugs (33%), with only 5% of physicians using dexmedetomidine [26]. However, these data, published a few years ago, may not accurately represent current sedation practices, as more recent publications reveal an increase in the use of dexmedetomidine [27–30], probably due to its anxiolytic, sedative and analgesic effects without affecting the respiratory pattern, although they may cause bradycardia and hypotension [29–32]. In our study, 11 patients developed bradycardia secondary to the use of dexmedetomidine, although none of the patients required intervention or interruption of the drug infusion. In contrast, IV benzodiazepines were the main agents responsible for respiratory depression events, suggesting that alternative approaches should be considered.

On the other hand, we observed that nonpharmacological measures to control discomfort were used by only two-thirds of the patients, and there was no relationship between the use of sedation and the use of these interventions. Milési et al. [33] suggested in a recent guideline for the management of bronchiolitis in the PICU that nonpharmacological strategies should be undertaken before administering sedatives, which seems to be a sensible approach.

The different behaviors of several clinical markers in children who received sedatives compared to those who did not should be highlighted; we observed that heart rate, the mWCAS, and the S/F ratio improved significantly more in the sedation group. Similarly, regarding the COMFORT-B scale scores, we also observed a significantly greater decrease in the number of children who received sedatives; after 6 h, the comfort scores were similar in both the sedation and non-sedation groups. These findings suggest that sedation may be helpful for tolerating NIV and could improve the success of an NIV trial. In terms of comfort and tolerability of this respiratory support, it has been reported a significant improvement with the use of neural triggering during NIV (NIV NAVA). It should be indicated that no patients receiving NIV NAVA were included in the present study [34].

In the present sample, less than 5% of patients were intubated, with a NIV success rate of 95.6%, which is higher than that reported in previous pediatric studies, where this rate ranged between 64 and 85% [2–5, 7, 21, 25]. Notably, in our study, 9 children who required intubation during the first 2 h of NIV were excluded; we concluded that due to the inherent severity of their condition and the short duration, it would not be possible to assess the effects of sedation and establish a causal link with treatment failure. Even considering the early failure of these patients, our success rate is much greater than that reported in the literature (93.8%). The availability of pediatric interfaces designed specifically for infants [35] and the greater experience acquired by physicians in NIV management, including rigorous patient selection, are factors that could have contributed to improving the success rate of NIV. Nonetheless, it cannot be ruled out a potential influence of an earlier institution of the technique in a less severe condition due to the current familiarity with NIV.

Notably, according to the baseline data (lower S/F ratio and higher heart rate and PRISM III score at NIV initiation), the sedation group seemed to have a significantly more severe condition than did the non-sedation group. This finding may agree with the hypothesis put forward by Leboucher et al., who stated that patients in the most severe condition are the most uncomfortable or that at least they are perceived as such [9]. However, multivariate analysis did not include the use of sedatives in the predictive model for the need for intubation, demonstrating that only the PRISM III score at NIV initiation and respiratory rate at 3 h were included in the final model. Interestingly, when the S/F ratio was included in the multivariate analysis, the S/F ratio at 3 h and PRISM III score at NIV initiation were the only factors independently linked to NIV failure. All these variables had already been identified as independent predictors of NIV failure in previous studies [3, 21, 25], even though PRISM III had not been calculated at NIV initiation before.

The PICU LOS was greater in children who received sedation than in those who did not, but these findings were not confirmed with the adjusted analysis. Muriel et al. observed a longer ICU stay in adults on NIV who received sedatives, although the authors did not perform multivariate analyses to support their findings [10]. Many factors may influence the length of stay in the PICU, especially the need for IMV and severity status [7, 8, 36], as also demonstrated by our results.

Among the limitations of the study, we highlight that the observational, noninterventional design, without a drug dosing protocol, made it difficult to assess the effects of each particular sedative. It is necessary to carry out randomized clinical trials to evaluate the effectiveness of each drug during NIV. Similarly, the effect of different non-pharmacological strategies could not be assessed. Also, the aforementioned low intubation rate compels us to interpret the present results cautiously. Lastly, the lack of a systematic evaluation of blood gases in all patients at the beginning of and during NIV limited the analysis of acidosis and hypercapnia as possible predictors of failure.

This study has several strengths. First, SEDANIV is the only multicenter study published to date that offers data on the management of patients receiving NIV; this topic has not yet been explored in children. Second, these results reflect the daily clinical practice of PICUs at different levels of care in Spain, so the results could be extrapolated to other centers with similar protocols and materials. Third, the prospective design, which included a large cohort of children, allowed us to evaluate a series of physiological parameters and clinical scales collected at regular intervals and in real time during the use of NIV.

## Conclusions

In agitated children less than five years of age under NIV, use of sedatives seems to be beneficial in terms of improving clinical markers and tolerance of the technique. Despite being more tachypneic and more hypoxemic and having a higher PRISM III score at NIV initiation, children who received sedatives had a significantly greater degree of comfort than did those who were not sedated, reaching similar COMFORT-B scale scores at the six-hour mark. Furthermore, heart rate, the S/F ratio, and the mWCAS improved significantly more in the treated group than in non-sedation group, without this being associated with greater NIV failure or a longer stay in the PICU. Further studies should focus on the ideal drugs, route of administration and dosing during NIV in children.

### Supplementary Information

Supplementary Material (DOCX 225 kb)

## Data Availability

The datasets used and analyzed during the current study are available from the corresponding author upon reasonable request.

## Abbreviations

ARF: Acute respiratory failure
AUC: Area under the ROC curve
BLPAP: Bilevel positive airway pressure
CPAP: Continuous positive airway pressure
EPAP: Expiratory positive airway pressure
HR: Hazard ratio
IMV: Invasive mechanical ventilation
IPAP: Inspiratory-positive airway pressure
IQR: Interquartile range
LOS: Length of stay
mWCAS: Modified Wood Clinical Asthma Scale
NIV: Noninvasive ventilation
OR: Odds ratio
PICU: Pediatric Intensive Care Unit
PRISM: Pediatric Risk of Mortality
ROC: Receiver operating characteristic
SD: Standard deviation
