# Supplementary material for: Implications of sedation during the use of noninvasive ventilation in children with acute respiratory failure (SEDANIV Study)
Source: Crit Care. 2024 Jul 11;28:235. doi: 10.1186/s13054-024-04976-2 (PMC11241858; doi:10.1186/s13054-024-04976-2)
Supplement: Supplementary file 1 — Supplementary Material (DOCX 225 kb) [file 13054_2024_4976_MOESM1_ESM.docx]

|  | Second-level PICU; *n=4* | Third-level PICU; *n=9* |
| --- | --- | --- |
| Type of PICU  Pediatric PICU  Mixed neonatal and pediatric PICU  Mixed adult and pediatric PICU | 3  1  0 | 9  0  0 |
| Bed capacity | 6 - 8 | 9 - 16 |
| Number of admissions per year | 200 - 400 | ≥ 500 |
| PICU admitting post-surgical patients | 4 | 9 |
| PICU admitting post-cardiac surgery | 0 | 8 |
| ECMO availability | 0 | 6 |
| Bed-to-nurse ratio for NIV patient care  Ratio 2:1  Ratio 3:1 | 4  0 | 7  2 |

**Supplementary material 1.** Characteristics of participating Pediatric Intensive Care Units

*PICU* Pediatric Intensive Care Unit; *ECMO* Extracorporeal membrane oxygenation; *NIV* noninvasive ventilation

**Supplementary material 2.** Differences in the use of sedatives in each participating hospital, which are numbered from 1 to 13.

**
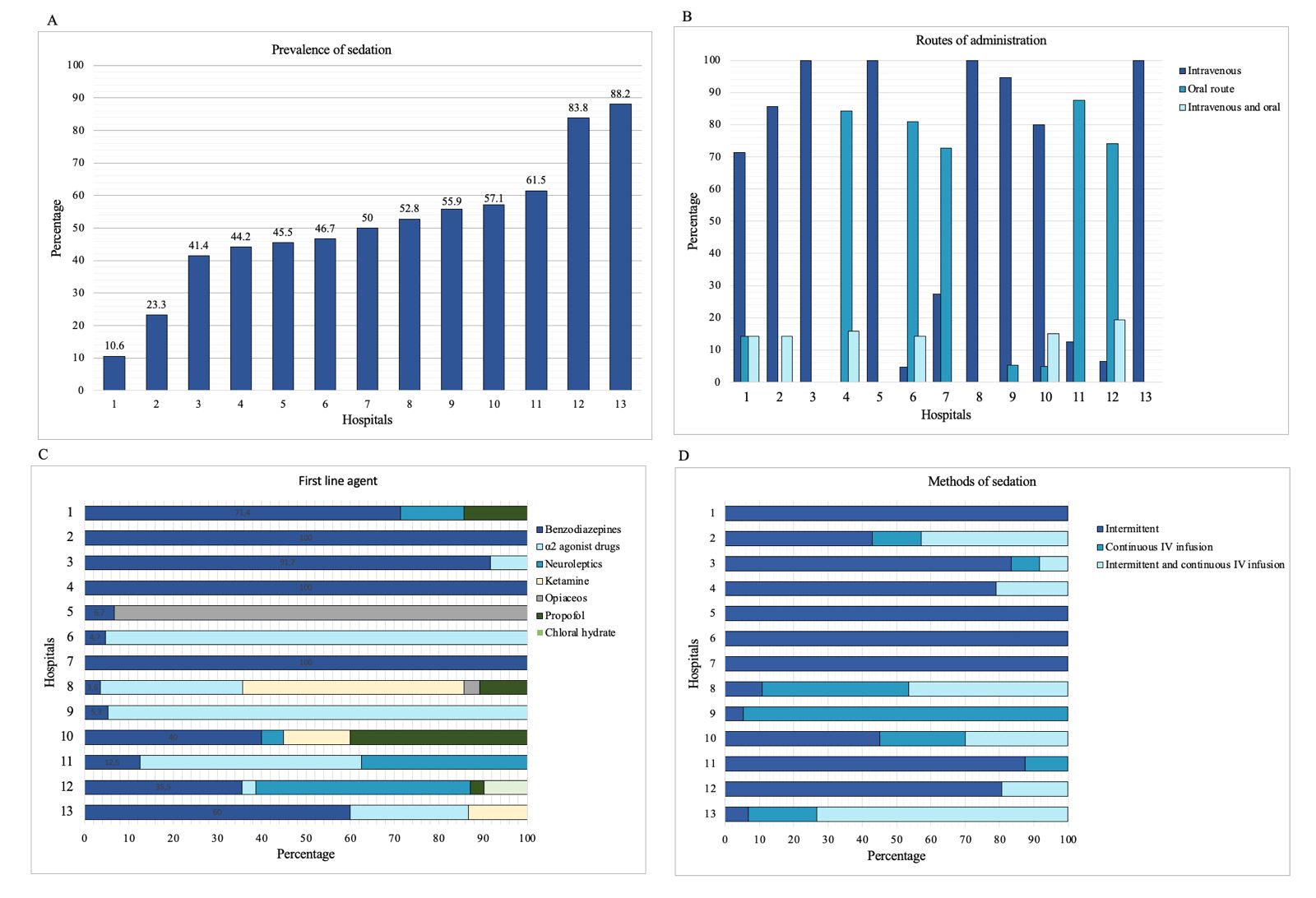
**


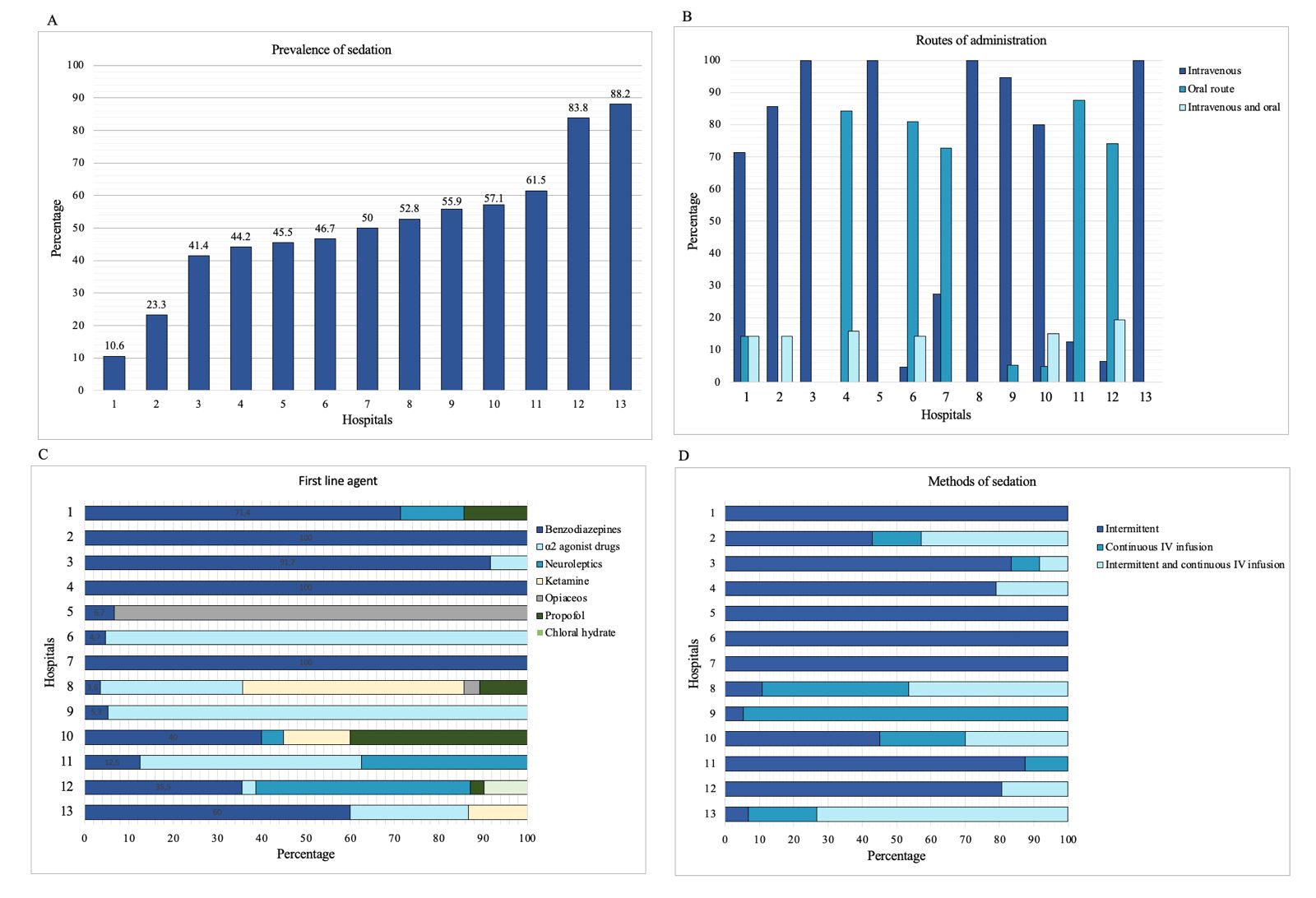


|  |  | H 0 | H 3 | H 6 | H 12 | H 24 | H 48 |
| --- | --- | --- | --- | --- | --- | --- | --- |
| Heart rate (beats/min)  Mean (SD) | NIV success  NIV failure  *p*-value | *n=457*  166.2 (24.7)  170.2 (32.3)  0.447 | *n=450*  147.3 (22.4)  148.8 (29.6)  0.822 | *n=443*  142.9 (21.4)  146.5 (34.6)  0.687 | *n=426*  139.8 (22.8)  142.8 (32.5)  0.520 | *n=371*  135.1 (21.5)  143.1 (30.4)  0.728 | *n=260*  131.6 (19.7)  128.6 (33.3)  0.795 |
| Respiratory rate (breaths/min)  Mean (SD) | NIV success  NIV failure  *p*-value | *n=457*  52.7 (13.9)  62.6 (17)  0.019 | *n=450*  46.8 (12.8)  55.4 (14.4)  0.016 | *n=443*  45.6 (12.8)  49.9 (12.3)  0.178 | *n=422*  44.3 (12.5)  60.6 (13.2)  <0.001 | *n=369*  42.4 (11.5)  56.3 (15)  0.007 | *n=271*  41.2 (10.9)  55.4 (24)  0.110 |
| S/F ratio  Median (IQR) | NIV success  NIV failure  *p*-value | *n= 244*  254.1 [194 – 323.3]  211 [151.7 – 310]  0.060 | *n= 268*  274.3 [235 – 368]  213.3 [171 – 277.1]  0.008 | *n= 262*  313.3 [237.5 – 372]  204.4 [170.5 – 245]  0.003 | *n= 256*  323.3 [242.5 – 388]  201.1 [157 – 240.6]  0.001 | *n= 224*  364 [274.3 – 441]  160 [143.3 – 261.7]  0.002 | *n= 167*  380 [277.1 – 456]  192 [191 – 202.7]  0.005 |
| mWCAS  Median (IQR) | NIV success  NIV failure  *p*-value | *n=388*  6 [5 – 7]  7 [6 – 9]  0.064 | *n=376*  5 [4 – 6]  6 [3 – 6.5]  0.086 | *n=366*  4 [3 – 6]  6 [4.5 – 7.3]  0.015 | *n=348*  4 [3 – 5]  6 [5 – 7]  <0.001 | *n=305*  3 [2 – 5]  5 [4 – 8.5]  0.024 | *n=212*  3 [2 – 4]  5 [4.5 – 6]  0.063 |
| COMFORT-B scale  Median (IQR) | NIV success  NIV failure  *p*-value | *n=374*  22 [18 – 24]  23[17 – 25]  0.577 | *n=358*  18 [13 – 21]  13.5 [11 – 21]  0.086 | *n=356*  16 [13 – 20]  14.5 [11.3 – 19.8]  0.372 | *n=347*  16 [12 – 18]  15.5 [11.5 – 18]  0.757 | *n=302*  15 [13 – 18]  14 [11.5 – 15.5]  0.255 | *n=212*  15 [13 – 18]  16.5 [14.5 – 17]  0.620 |
| If Bi-level pressure  IPAP (cmH2O)  Median (IQR) | NIV success  NIV failure  *p*-value | *n=361*  10 [9 – 12]  11 [8 – 15]  0.239 | *n=337*  10 [9 – 12]  12 [8 – 15]  0.009 | *n=345*  10 [9 – 12]  14 [8 – 15]  0.002 | *n=331*  10 [9 – 12]  14 [12 – 17]  <0.001 | *n=281*  10 [9 – 12]  13.5 [12 – 17]  0.005 | *n=331*  10 [9 – 12]  13 [11 – 14]  0.133 |
| If Bi-level pressure  EPAP (cmH2O)  Median (IQR) | NIV success  NIV failure  *p*-value | *n=361*  6 [5 – 6]  6 [5 – 8.3]  0.092 | *n=378*  6 [5 – 6]  7 [6 – 9]  0.001 | *n=378*  6 [5 – 6]  6 [5 – 8.3]  0.005 | *n=331*  6 [5 – 6]  6.5 [6 – 9]  0.009 | *n=281*  6 [5 – 6]  6.5 [6 – 9]  0.028 | *n=331*  6 [5 – 6]  7 [6.5 – 7]  0.079 |
| CPAP (cmH2O)  Median (IQR) | NIV success  NIV failure  *p*-value | *n=96*  5 [5 – 6]  6 [6 – 6]  0.415 | *n=78*  5 [5 – 6]  5.5 [5 – 6]  0.966 | *n=74*  5 [5 – 6]  5.2 [4.5 – 6]  0.514 | *n=72*  5 [5 – 6]  4.8 [4 – 6]  0.426 | *n=87*  5 [5 – 6]  - | *n=57*  6 [5 – 6]  - |
| Tidal volume (ml/Kg)  Median [IQR] | NIV success  NIV failure  *p*-value | *n=304*  8.7 [7 – 10]  8 [6 – 10]  0.519 | *n=377*  6 [5 – 7]  7 [6 – 9]  0.001 | *n=371*  6 [5 – 7]  6.5 [6 – 9]  0.015 | *n=282*  9 [8 – 11]  8.5 [7 – 10]  0.319 | *n=256*  9 [8 – 11]  8.5 [6.9 – 9.6]  0.482 | *n=180*  9 [7 – 10]  10 [7.3 – 12.5]  0.501 |
| pH  Median (IQR) | NIV success  NIV failure  *p*-value | *n=284*  7.33 [7.27 – 7.38]  7.30 [7.17 – 7.36]  0.199 | *n=86*  7.35 [7.31 – 7.40]  7.35 [7.29 – 7.38]  0.434 | *n=86*  7.35 [7.32 – 7.39]  7.30 [7.21 – 7.37]  0.066 | *n=93*  7.38 [7.33 – 7.4]  7.25 [7.22 – 7.31]  0.004 | *n=106*  7.38 [7.34 – 7.41]  7.34 [7.29 – 7.35]  0.204 | *n=61*  7.39 [7.35 – 7.42]  7.30 [7.22 – 7.30]  0.002 |
| pCO2 (mmHg)  Median (IQR) | NIV success  NIV failure  *p*-value | *n=284*  47 [39 – 61]  54 [43 – 65.8]  0.233 | *n=86*  45 [38.5 – 55]  55 [44 – 57]  0.242 | *n=86*  43.5 [37 – 54]  60.2 [47 – 69]  0.004 | *n=93*  44 [38.4 – 49]  63.2 [58 – 73]  0.004 | *n=106*  44.5 [38.6 – 53.5]  59 [51.5 – 66]  0.011 | *n=61*  46.3 [38.4 – 53]  62.3 [60.2 – 86.5]  0.004 |

**Supplementary material 3.** Data collected during the first 48 hours of NIV for the two cohorts of patients (success group vs failure group).

*S/F ratio SpO2/FiO2 ratio; NIV* noninvasive ventilation; *mWCAS* modified Wood’s Clinical Asthma Score; *PRISM III score* Pediatric Risk of Mortality Score III.

**Supplementary material 4.**  Risk factors related to NIV failure. Summary of the multivariate logistic regression models.

|  | | B | Odds ratio (95% CI) | | | *p value* |
| --- | --- | --- | --- | --- | --- | --- |
| PRISM score at NIV initiation  Respiratory rate at 3 hours | | 0.342  0.042 | 1.408 (1.230 – 1.611)  1.043 (1.009 – 1.079) | | | *<0.001*  *0.014* |
| AUC = 0.807 (95% CI 0.687 – 0.928); *p=<0.001*  Good predictive power (-2LL=129.57). The Hosmer-Lemeshow test (calibration): p= 0.334.  Cutoff value of PRISM score: 4.2 (Sensitivity 80 % and Specificity 81.6 %)  Cutoff value of Basal respiratory rate: 79 breaths/min (Sensitivity 80 % and Specificity 81.6%) | | | | | | |
| ^b^Multivariate analysis including SF ratio *(n=262*) | | | | | | |
|  | B | | | Odds ratio (95% CI) | *p value* | |
| S/F ratio at 3 hours  PRISM score at NIV initiation | -0.008  0.368 | | | 0.992 (0.984 – 0.999)  1.445 (1.215 – 1.719) | *0.038*  *<0.001* | |
| AUC = 0.815 (95% CI 0.691 – 0.939); *p=<0.001*  Good predictive power (-2LL=86.19). The Hosmer-Lemeshow test (calibration): p= 0.449.  Cutoff value of S/F ratio: 180.5 (Sensitivity 73.3% and Specificity 72%) | | | | | | |

**^a^Multivariate analysis of the general sample**

*NIV* Non-invasive ventilation, *PRISM III score* Pediatric Risk of Mortality Score III, *S/F ratio* SpO2/FiO2 ratio. *AUC* Area under the ROC (Receiver operating characteristic) Curve.

^a^ Variables included in the analysis: Sedation (Not=0 or Yes=1), PRISM score at NIV initiation, respiratory rate at 3 hours.

^b^ Variables included in the analysis: Sedation in the first 3 hours of NIV (Not or Yes), S/F ratio at 3 hours, PRISM score at NIV initiation. SpO2 over 97 % were excluded to calculate SF ratio.

**Supplementary material 5.** Risk factors related to longer length of PICU stay. Univariate and multivariate Cox regression analysis.

|  | | **Univariate analysis** | | | **Multivariate analysis** | | | |
| --- | --- | --- | --- | --- | --- | --- | --- | --- |
|  | **HR** | | **95% CI** | ***p value*** | | **HR** | **95% CI** | ***p* value** |
| Sedation at any time during NIV | 0.849 | | 0.704 – 1.023 | *0.085* | | 0.997 | 0.783 – 1.269 | *0.980* |
| Age (months) | 1.014 | | 1.008 – 1.021 | *<0.001* | | 0.994 | 0.976 – 1.013 | *0.559* |
| Weight (kg) | 1.050 | | 1.030 – 1.070 | *<0.001* | | 1.072 | 1.041 – 1.103 | *<0.001* |
| Underlying disease | 0.528 | | 0.408 – 0.685 | *<0.001* | | 0.781 | 0.560 – 1.090 | 0.146 |
| PRISM score at NIV initiation | 0.921 | | 0.885 – 0.960 | *<0.001* | | 1.048 | 0.988 – 1.112 | *0.118* |
| PRISM score at 24 H | 0.862 | | 0.817 – 0.910 | *<0.001* | | 0.859 | 0.803 – 0.920 | *<0.001* |
| Respiratory rate (breaths/min) at 3 hours | 0.987 | | 0.980 – 0.994 | *<0.001* | | 0.999 | 0.986 – 1.012 | *0.870* |
| Respiratory rate (breaths/min) at 6 hours | 0.990 | | 0.983 – 0.998 | *0.010* | | 1.002 | 0.991 – 1.014 | *0.681* |
| Respiratory rate (breaths/min) at 12 hours | 0.988 | | 0.981 – 0.995 | *0.001* | | 1.017 | 1.006 – 1.027 | *0.001* |
| FiO2 (%) at NIV initiation | 0.584 | | 0.336 – 1.016 | *0.057* | | 0.541 | 0.229 – 1.278 | *0.161* |
| FiO2 (%) at 3 hours | 0.294 | | 0.129 – 0.671 | *0.004* | | 1.327 | 0.112 – 15.75 | *0.823* |
| FiO2 (%) at 6 hours | 0.264 | | 0.103 – 0.679 | *0.006* | | 4.856 | 0.734 – 32.14 | *0.101* |
| FiO2 (%) at 12 hours | 0.136 | | 0.050 – 0.365 | *<0.001* | | 0.031 | 0.004 – 0.219 | *<0.001* |
| SpO2 (%) at NIV initiation | 1.025 | | 0.999 – 1.052 | *0.063* | | 0.966 | 0.924 – 1.010 | 0.124 |
| SpO2 (%) at 3 hours | 1.047 | | 1.014 – 1.080 | *0.004* | | 1.069 | 1.023 – 1.117 | *0.003* |
| SpO2 (%) at 6 hours | 1.040 | | 1.005 – 1.076 | *0.026* | | 1.005 | 0.947 – 1.067 | 0.867 |
| SpO2 (%) at 12 hours | 1.038 | | 0.998 – 1.079 | *0.060* | | 0.996 | 0.942 – 1.053 | 0.879 |
| mWCAS at 6 hours | 0.939 | | 0.887 – 0.994 | *0.030* | | 1.098 | 0.953 – 1.266 | 0.196 |
| mWCAS at 12 hours | 0.906 | | 0.857 – 0.957 | *<0.001* | | 0.935 | 0.872 – 1.003 | 0.060 |
| NIV failure | 0.314 | | 0.194 – 0.508 | *<0.001* | | 0.275 | 0.130 – 0.580 | *<0.001* |
| Duration of NIV (hours) | 0.890 | | 0.858 – 0.923 | *<0.001* | | 0.995 | 0.993 – 0.997 | *<0.001* |

*NIV* noninvasive ventilation; *mWCAS* modified Wood’s Clinical Asthma Score; *PRISM III score* Pediatric Risk of Mortality Score III.
